# Special Section Guest Editorial: Introduction to the Special Section Celebrating 30 years of Open Source Monte Carlo Codes in Biomedical Optics

**DOI:** 10.1117/1.JBO.27.8.083001

**Published:** 2022-08-08

**Authors:** Qianqian Fang, Fabrizio Martelli, Lothar Lilge

**Affiliations:** a Northeastern University, Boston, Massachusetts, United States; b University of Florence, Florence, Italy; c University of Toronto, Toronto, Ontario, Canada

## Abstract

The editorial introduces the JBO Special Section Celebrating 30 Years of Open Source Monte Carlo Codes in Biomedical Optics for Volume 27, Issue 8.

This special section of the *Journal of Biomedical Optics* (Volume 27, Issue 8) celebrates 30 years since the first posting of a program called mcml.c (Monte Carlo multi-layered), more commonly known as MCML. The code’s history is presented in the commentary by Prof. Steven Jacques.^1^ The impact of the MCML code on almost all areas of biomedical optics cannot be overstated.

During the nine years, starting with the first report using Monte Carlo (MC) simulations to describe light distribution in biological tissue by Wilson and Adam in 1983^2^ until the publication of the MCML code in 1992, only a small number of groups had employed MC techniques, resulting in 19 publications. The field started to expand rapidly with over 100 publications in 3 years, from posting the code to the 1995 paper by Wang, Jacques, and Zheng.^3^ In the next seven years, this increased to 700 citations and has since settled at about 200 publications per year reporting the use of MC simulations as part of the presented research. The constant current reference to MCML or any of the other newer codes is a reflection of biophotonics as a whole representing a mature technology being applied to a wide range of biomedical problems, rather than biophotonics and light propagation in turbid media being the principal target of the investigation.

Given the lack of analytical solutions in determining the photon budget or distribution for the vast majority of medical diagnostic and therapeutic photonic applications, generally applicable, faster, and easily accessible MC software has seen a constant demand from the community, reflected amongst many of the works included in this special section. A subset of the papers focuses on adapting MC methods or codes for particular biophotonics techniques, enabling researchers to explore additional imaging capabilities in an efficient fashion. Articles related to this research domain include the work by Pattelli and Mazzamuto,^4^ as well as that from Fu and Richards,^5^ focusing on the propagation of ultrafast pulses and age-related changes in diffuse optical properties as function of age, respectively.

Lastly, to facilitate the rapidly emerging machine-learning and deep-learning (DL) techniques related to biophotonics, the development of rich datasets for *in silico* phantoms and data depositories for training and cross-validation is another trend.

While novice users can start with well-established and validated MC codes, such as MCML or MCX and its derivatives, using novel accelerated or specifically adapted MC codes may not give the same confidence pertaining to their expected accuracy, particularly if code validation steps were not explicitly provided. In work by Sassaroli et al.,^6^ a method for code verification has been proposed. The method can be applied by the user and relies on analytical comparisons to verify the average photon trajectory and the reflection/refraction conditions at object boundaries.

New open-source MC simulators are presented by Hayakawa et al.,^7^ Gröhl et al.,^8^ and Zhang and Fang,^9^ offering user-friendly interfaces and versatile MC simulations in 3D heterogeneous media for general purpose use or modality-specific applications, such as photoacoustic imaging.

Acceleration of MC execution is presented either through hardware implementation—see Yan et al.^10^ for polarized light transport—or cloud computing, as shown by Fang and Yan^11^ and Wang et al.^12^ for the MCX and FullMonte codes respectively.

An alternative approach for reduced MC execution times tackles the photon statistics-based noise for limited photon packages and proposes DL approaches as added variance reduction schemes proposed by Raayai Ardakani et al.^13^ The proposed DL denoising approach can result in an over 20-fold reduction in required photon packages to yield similar results. The use of a stochastic Gauss–Newton method was demonstrated to be beneficial in reducing the photon package numbers when solving iterative inverse problems such as image formation in photoacoustic applications by Hänninen et al.^14^

Accurately considering complex tissue boundaries in MC simulation has been a vital community interest. A novel shape representation using a signed distance function is explored by McMillan, Bruce, and Dholakia.^15^ Similarly, the variability of optical properties remains a challenge to the field and the work by Kao and Sung,^16^ employing CW spectroscopy and anatomically correct MC models to quantify personalized properties values, presents an interesting approach with direct translation for photothermal and photodynamic therapies.

Next to the code mentioned above for MC simulations of polarized light propagation in turbid media,^10^ two approaches to simulate photoacoustic images are presented. Gröhl et al.^8^ used Python architecture to generate photoacoustic images using a built-in library of biological structures, as well as the above-mentioned work by Hänninen et al.^14^

Simulation of time-domain diffuse correlation spectroscopy provides a challenging environment, as stochastic photon path simulations need to be paired with the wave character of light to predict the speckle intensity fluctuations correlated with blood flow. This code is made publicly available by Cheng et al.^17^ It would be interesting to see a convergence of that code with the work by Fu and Richards^5^ mentioned above.

Two articles focus on MC simulations for photodynamic therapy (PDT) treatment planning. One of those describes a retrospective study of patient eligibility by Li et al.,^18^ which demonstrated that more patients could benefit from the described antimicrobial therapy; the other, by S. Wang et al.,^12^ demonstrates that interstitial PDT can provide personalized fiber optical source placement and the optical power delivered to each of these sources based on an iterative elimination process.

Similar to the speckle intensity fluctuation prediction code by Cheng et al.^17^ using MC and wave formalism mentioned above, the code by Jafari et al.^19^ aims to predict the tomographic blood flow from the measured speckle pattern images, employing a gradient descent with adaptive learning to minimize the required forward MC simulations.

An article by Fine, McShane, and Coté^20^ demonstrates the utility of MC simulations to improve the performance and lifetime of implantable optical biosensors, in particular for glucose sensing.

With the push towards machine and deep learning also in biophotonics, there is the need for improved ease in model generations, as attainable by BlenderPhotonics software^9^ mentioned above and general public databases for at least standard geometries. Bürmen, Pernuš, and Naglič^21^ present the framework for an open dataset providing reflectance, transmittance, and absorbed energy for a range of tissues, sources, and detectors.

How the need for generating a large dataset for machine and deep learning approaches in biophotonics could be satisfied by available open-source, cloud-based MC software MCX and others is demonstrated by Nizam et al.,^22^ rounding out the range of research presented in this collection.

It is evident that increasing utilization of cloud computing, generation of large publicly shared *in silico* phantoms, and computational outputs for a range of 2D and 3D biophotonics applications will become an integral part of biomedical optics. The various general and specialized MC codes, including those presented here, paired with further variance reduction schemes, are well positioned to become the primary workhorses to address the community’s challenges.

We would like to thank all contributors, including Pattelli and Mazzamoto,^4^ for the artwort presented in the issue cover, and reviewers, as well as the JBO staff for the successful completion of this special section. Here’s to the next 30 years of MC in biophotonics!
